# Tripterygium Glycoside Tablets Combined With Conventional Synthetic Disease-Modifying Antirheumatic Drugs for Treating Rheumatoid Arthritis: Protocol for a Prospective, Multicenter, Open-Label Randomized Controlled Trial

**DOI:** 10.2196/87156

**Published:** 2026-04-27

**Authors:** Liu Lv, Tuoran Wang, Jing Lv, Chunping Liu, Luming Zhao, Yu Zhou

**Affiliations:** 1 Department of Rheumatology, Dongzhimen Hospital Affiliated to Beijing University of Chinese Medicine, Beijing, China; 2 Acupuncture and Moxibustion Hospital, Institute of Acupuncture and Moxibustion, China Academy of Chinese Medical Sciences, No. 16 Nanxiaojie, Dongzhimennei, Dongcheng District, Beijing, China, 86 13520302630; 3 Department of Rheumatology, Wuhan Hospital of Traditional Chinese Medicine, Wuhan, China

**Keywords:** rheumatoid arthritis, Tripterygium glycoside tablets, conventional synthetic disease-modifying antirheumatic drugs, csDMARDs, clinical trials, complementary medicine, randomized controlled trial

## Abstract

**Background:**

Tripterygium glycoside tablets (TGTs), a traditional Chinese medicine–based therapy and a type of conventional synthetic disease-modifying antirheumatic drug (csDMARD), have shown promise as a cost-effective alternative for rheumatoid arthritis (RA). However, there is limited evidence regarding the most effective combinations with other csDMARDs, such as methotrexate, leflunomide, and hydroxychloroquine. This study evaluates the 12-week efficacy and safety of TGT-based regimens in patients with moderately active RA.

**Objective:**

This study aims to evaluate the clinical efficacy and safety of the csDMARD combination regimens mainly based on TGTs in the treatment of RA, using multicenter clinical data, and to explore the clinical characteristics of TGTs and identify the optimal combination therapy through a randomized controlled trial.

**Methods:**

This multicenter, open-label randomized controlled trial recruited 188 participants (47 per group) from 3 hospitals. Eligible patients were stratified by study center and randomized in a 1:1:1:1 ratio to receive TGT monotherapy, TGT plus methotrexate, TGT plus leflunomide, or TGT plus hydroxychloroquine. The primary outcome was the American College of Rheumatology 20% improvement response rate. Secondary outcome measures included the Disease Activity Score-28 (DAS28) using erythrocyte sedimentation rate and DAS28 using C-reactive protein response rates, the Clinical Disease Activity Index, the Simplified Disease Activity Index, the pain visual analog scale (0 to 10 points), the patient global assessment, the medical doctor global assessment, quality of life (36-Item Short Form Health Survey), and the Health Assessment Questionnaire Disability Index. All enrolled patients were followed up every 4 weeks for a total of 12 weeks. Adverse events were recorded during the observation period of the study. All patients randomized in this study will be included in the intention-to-treat analysis.

**Results:**

This study was funded in February 2024. Funding was provided by the Beijing Tongzhou District Science and Technology Planning Project. Recruitment of participants commenced on September 1, 2024, and concluded on December 31, 2025. A total of 181 patients were enrolled. Final data collection has been completed, and data cleaning is currently underway. Statistical analyses have not yet been performed. The primary results are expected to be submitted for publication by the end of 2026.

**Conclusions:**

This trial uses multicenter clinical data to provide robust evidence on the efficacy and safety of TGTs in combination with csDMARDs for the treatment of RA with moderate disease activity.

## Introduction

Rheumatoid arthritis (RA) is a chronic autoimmune disease characterized by synovial inflammation and progressive joint destruction, leading to significant disability and reduced quality of life [1-3]. Globally, RA affects approximately 0.5% to 1% of the population [3-5], with a prevalence of 0.28% to 0.45% in China, affecting more than 5 million individuals [6,7]. The disease not only imposes a heavy burden on patients and their families but also places significant economic pressure on health care systems due to the need for long-term treatment and management of complications [7,8].

Despite significant progress in RA therapy, including the development of conventional synthetic disease-modifying antirheumatic drugs (csDMARDs), biologic disease-modifying antirheumatic drugs (bDMARDs), and targeted synthetic disease-modifying antirheumatic drugs (tsDMARDs), achieving remission remains a challenge [9-11]. Additionally, although bDMARDs and tsDMARDs are highly effective and can be used as monotherapy in certain cases, their cost-effectiveness compared with csDMARDs is still questionable. While bDMARDs and tsDMARDs have improved outcomes for many patients, their high cost and associated safety risks—such as increased susceptibility to infections, malignancies, and thromboembolic events—limit their accessibility, particularly in low- and middle-income countries [12-14].

In this context, Tripterygium glycoside tablets (TGTs), a traditional Chinese medicine–based therapy and a type of csDMARD, have emerged as a promising alternative [15]. TGTs have shown comparable efficacy to methotrexate in monotherapy and potential synergistic effects when combined with other csDMARDs [15-19]. However, robust evidence on the optimal combination strategy, safety profile, and efficacy of TGTs in patients with moderate disease activity remains scarce [17]. This subgroup of patients is often underrepresented in clinical trials but represents a significant proportion of real-world RA cases.

This trial aims to address these critical gaps by evaluating the 12-week efficacy and safety of TGTs combined with methotrexate, leflunomide, or hydroxychloroquine in a well-diagnosed population with RA. By focusing on short-term outcomes, the study seeks to identify the most effective and safest TGTs-based regimen, providing evidence to guide clinical practice and improve treatment outcomes for patients with RA globally.

## Methods

### Study Design

**Figure 1.**
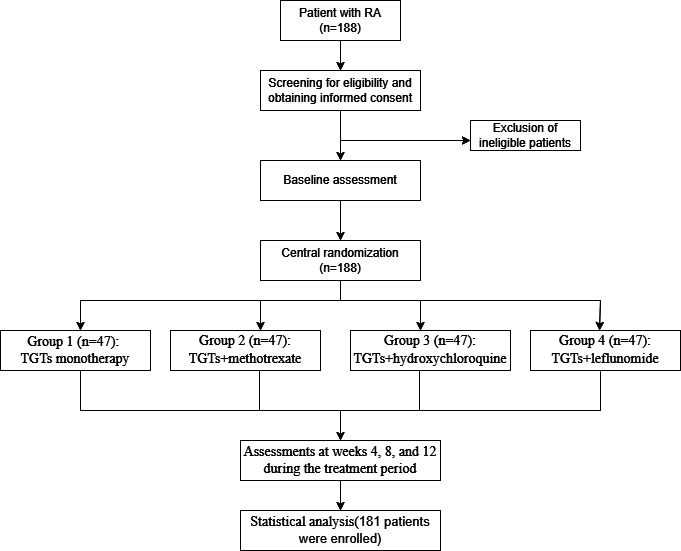
Flowchart of the randomized controlled trial. RA: rheumatoid arthritis; TGT: Tripterygium glycoside tablet.

This is a prospective, multicenter, open-label randomized controlled trial (RCT) designed to evaluate and generate evidence on the optimal combination of TGTs with other csDMARDs (methotrexate, leflunomide, and hydroxychloroquine). The trial is being conducted at 3 sites in China: Dongzhimen Hospital, Beijing University of Chinese Medicine; Acupuncture and Moxibustion Hospital, China Academy of Chinese Medical Sciences; and Wuhan Hospital of Traditional Chinese Medicine. Eligible patients with RA were enrolled and assigned via block randomization using SAS (version 9.3; SAS Institute). Participants were allocated in a 1:1:1:1 ratio to 1 of 4 treatment groups: group 1 (TGT monotherapy), group 2 (TGTs+methotrexate), group 3 (TGTs+hydroxychloroquine), and group 4 (TGTs+leflunomide). The total observation period was 12 weeks, including a 4-week treatment period, an 8-week treatment period, and a 12-week treatment period. The flowchart of this study is shown in Figure 1, and the evaluation time points are shown in Textbox 1.

Textbox 1. **Eligibility criteria for the study.**

**Inclusion criteria**

- Aged 18-65 years
- Disease Activity Score-28: >3.2 and ≤5.1
- Men or women without reproductive requirements
- No participation in any clinical trial within 4 weeks before enrollment
- Provision of written informed consent

**Exclusion criteria**

- Presence of serious organ disease or mental disease
- Inability to adhere to medication for 12 weeks due to any reason
- Diagnosis of Sjögren syndrome, ankylosing spondylitis, systemic lupus erythematosus, dermatomyositis, or other rheumatic diseases
- Renal and hepatic insufficiency
- Pregnancy or lactation (breastfeeding)
- Severe gastrointestinal ulcer or active gastrointestinal bleeding
- Use of glucocorticoids or biological agents within 4 weeks before enrollment

### Setting and Participants

Patients who met the 1987 American College of Rheumatology (ACR) [20] or the 2010 ACR/European League Against Rheumatism [21] classification criteria for RA were eligible for inclusion. The detailed inclusion and exclusion criteria are provided in Textbox 1.

### Recruitment Process

Participants were recruited primarily from the Department of Rheumatology at Dongzhimen Hospital of Beijing University of Chinese Medicine, the Department of Rheumatology at Acupuncture and Moxibustion Hospital at China Academy of Chinese Medical Sciences, and the Department of Rheumatology at Wuhan Hospital of Traditional Chinese Medicine, where research assistants assisted in screening participants. Additional participants were recruited through advertisements such as job postings.

### Randomization, Allocation Concealment, and Blinding

Eligible patients with RA were enrolled and assigned to 4 treatment groups using block randomization with a 1:1:1:1 allocation ratio. Statistical experts who were not involved in the study or data analysis generated the block randomization sequence using the SAS software.

This is an open-label study; therefore, neither researchers nor participants were blinded. Only adjudicators of outcome measures and statistical analysts were blinded to group allocation.

### Interventions

All participants received targeted TGT-based therapy, administered either as monotherapy or in combination with methotrexate (10‐15 mg/wk), leflunomide (10‐20 mg/d), or hydroxychloroquine (200‐400 mg/d). Participants were evaluated at baseline and then every 4 weeks for a total of 12 weeks.

For ethical reasons, nonsteroidal anti-inflammatory drugs (NSAIDs), such as diclofenac sodium enteric-coated tablets, were permitted as rescue therapy when pain became intolerable during the study. The permitted regimen was 25 mg orally 3 times daily before meals, and the use, dosage, and therapeutic effects were recorded in detail.

However, during the trial period, all other medications with similar mechanisms or effects to the study drug were prohibited, except for rescue therapy for intolerable RA pain. This included glucocorticoids (eg, prednisone acetate and methylprednisolone), biologic agents (eg, adalimumab and secukinumab), and other disease-modifying antirheumatic drugs (eg, cyclophosphamide and cyclosporine).

### Outcome Measures

#### Primary Outcome Measurement

The American College of Rheumatology 20% improvement (ACR20) response rate at week 12 is defined as ≥20% improvement in both tender joint count (TJC) and swollen joint count (SJC), as well as ≥20% improvement in at least 3 of the following 5 domains: patient global assessment (PGA), medical doctor global assessment (MDGA), patient pain visual analog scale (VAS), Health Assessment Questionnaire Disability Index (HAQ-DI), and acute-phase reactants (erythrocyte sedimentation rate [ESR] or C-reactive protein [CRP]).

#### Secondary Outcome Measurement

##### Overview

The secondary outcome measures included Disease Activity Score-28 (DAS28) using ESR (DAS28-ESR) and DAS28 using CRP (DAS28-CRP) response rates, Clinical Disease Activity Index (CDAI), Simplified Disease Activity Index (SDAI), pain VAS score (0 to 10), PGA, MDGA, quality of life (36-Item Short Form Health Survey [SF-36]), HAQ-DI, and inflammatory factor levels. The incidence of adverse events was measured using Common Terminology Criteria for Adverse Events (CTCAE), version 5.0. The measurement of each outcome and the schedule of visits are shown in Table 1. Participants were reminded to complete these diaries via text message, phone call, email, and instant messaging (WeChat; Tencent Holdings Ltd).

**Table 1.** Visit schedule and assessments.

Variables	Registration (visit 0)	Baseline (visit 1, day 0)	Week 4 (visit 2, days 25-31)	Week 8 (visit 3, days 49-63)	Week 12 (visit 4, days 74-94)
Enrollment					
Eligibility screen	✓				
Baseline demographic		✓			
Clinical characteristics		✓			
Informed consent		✓			
Randomization		✓			
Interventions					
Group 1: TGT^a^ monotherapy		✓	✓	✓	✓
Group 2: TGTs+methotrexate		✓	✓	✓	✓
Group 3: TGTs+hydroxychloroquine		✓	✓	✓	✓
Group 4: TGTs+leflunomide		✓	✓	✓	✓
Assessments					
Primary outcome: ACR20^b^ response rate			✓	✓	✓
Secondary outcome					
DAS28-ESR^c^		✓	✓	✓	✓
DAS28-CRP^d^		✓	✓	✓	✓
CDAI^e^		✓	✓	✓	✓
SDAI^f^		✓	✓	✓	✓
Pain VAS^g^		✓	✓	✓	✓
PGA^h^		✓	✓	✓	✓
MDGA^i^		✓	✓	✓	✓
Quality of life (SF-36^j^)		✓	✓	✓	✓
HAQ-DI^k^		✓	✓	✓	✓
Inflammatory factors		✓	✓	✓	✓
Safety monitoring					
Adverse events		✓	✓	✓	✓
Research completion					✓

a TGT: Tripterygium glycoside tablet.

b ASR20: American College of Rheumatology 20% improvement.

c DAS28-ESR: Disease Activity Score-28 using erythrocyte sedimentation rate.

d DAS28-CRP: Disease Activity Score-28 using C-reactive protein.

e CDAI: Clinical Disease Activity Index.

f SDAI: Simplified Disease Activity Index.

g VAS: visual analog scale.

h PGA: patient global assessment.

i MDGA: medical doctor global assessment.

j SF-36: 36-Item Short Form Health Survey.

k HAQ-DI: Health Assessment Questionnaire Disability Index.

##### DAS28-ESR and DAS28-CRP Response Rates

DAS28-ESR and DAS28-CRP response rates were used to determine RA disease activity, including the number of swollen joints (SJC; 0 to 28), the number of tender joints (TJC; 0 to 28), the PGA (VAS; 0 to 10), the ESR (mm/h), and the CRP (mg/L) level. The DAS28 activity rate was low (DAS28 ≤3.2). According to the European Alliance of Associations for Rheumatology (EULAR) response criteria, a change in DAS28 from baseline of >1.2 indicates an excellent response, a change of ≥0.6 to ≤1.2 indicates a moderate response, and a change of ≤0.6 indicates no response [22].

##### Clinical Disease Activity Index

The CDAI was calculated using the following formula: CDAI = TJC + SJC + PGA + MDGA. The scoring criteria are as follows: ≤2.8 indicates clinical remission, >2.8 to ≤10.0 indicates low disease activity, >10.0 to <22.0 indicates moderate disease activity, and >22 indicates high disease activity [23].

##### Simplified Disease Activity Index

The SDAI is an extended version of the CDAI that includes CRP. It is calculated using the following formula: SDAI = TJC + SJC + PGA + MDGA + CRP (mg/dL). The scoring criteria are as follows: ≤3.3 indicates clinical remission, >3.3 to ≤11.0 indicates low disease activity, >11.0 to ≤26.0 indicates moderate disease activity, and >26.0 indicates high disease activity [24].

##### Pain VAS Score

The VAS is a tool in which patients rate the intensity of their pain on a straight line ranging from 0 to 10 points. A score of 0 represents “no pain,” and a score of 10 represents “very severe pain.” The VAS reflects the subjective pain level of patients with RA and is often used to compare pain levels before and after treatment.

##### Patient Global Assessment

The PGA is an overall score (0 to 10) based on the patient’s subjective perception of their disease activity. It reflects the patient’s perception of the effect of treatment and is based on feelings of pain, fatigue, and functional limitations.

##### Medical Doctor Global Assessment

The MDGA is an overall score ranging from 0 to 10 based on the physician’s evaluation of the patient’s current disease activity. The score reflects the clinician’s judgment of the inflammatory burden and incorporates findings from the patient’s physical examination (eg, joint swelling and tenderness), laboratory results, and functional observations.

##### Quality of Life Assessment (SF-36)

The SF-36 covers 8 dimensions, such as physical function and social function, with a total of 36 items. Each dimension was standardized to a score of 0 to 100, and the total score reflected the overall quality of life. The long-term impact of RA on several dimensions of life was assessed.

##### Health Assessment Questionnaire

The HAQ-DI is a tool used to assess activities of daily living in individuals with RA. It contains 20 questions covering activities such as dressing, eating, and walking, reflecting the actual impact of the disease on daily life. The HAQ-DI is scored from 0 to 3, with 0 indicating no difficulty and 3 indicating inability to complete the activity. Higher total scores indicate more severe functional limitations.

##### Detection of Inflammatory Factors

These factors include tumor necrosis factor-α (TNF-α) and interleukin-6 (IL-6). TNF-α, a proinflammatory cytokine, can be detected using an enzyme-linked immunosorbent assay. Elevated levels of TNF-α indicate active inflammation. IL-6 mediates the acute-phase response and is associated with joint destruction. IL-6 is detected by the same methodology as TNF-α.

### Incidence of Adverse Events

Safety will be evaluated through routine blood tests, as well as renal and hepatic function tests.

Drug-related adverse events, including infections, liver dysfunction, kidney dysfunction, and treatment-related complications (eg, injection site reactions), were recorded. Serious adverse events were documented separately. Adverse events will be summarized by frequency (eg, number and percentage of participants affected) and severity according to the CTCAE criteria.

### Patients’ Compliance Assessment

Patients’ adherence was assessed by counting the number of remaining cartridges at the end of treatment. In addition, dropout status and reasons for discontinuation were recorded throughout the 12-week observation period.

### Patients and Public Involvement

Patients and the public were not involved in the development of the research question or study design. They will not participate in trial recruitment, conduct, or reporting of results. Trial results will be disseminated to participants via social media.

### Sample Size

The sample size was calculated for a multicenter RCT with 1 common control group (TGT monotherapy) and 3 active treatment groups (TGTs+methotrexate, TGTs+hydroxychloroquine, and TGTs+leflunomide). The primary hypothesis is that each combination therapy will be superior to TGTs monotherapy for the ACR20 response rate at week 12. The assumed ACR20 response rates were derived from an unpublished study conducted at our center in 2022 (Liu Lv, unpublished data, May 2022). This study included 30 patients with moderate RA (DAS28 3.2-5.1), randomized 1:1 to receive either TGT monotherapy (n=15) or TGTs+methotrexate (n=15) for 12 weeks. The observed ACR20 response rates were 53.3% (8/15) in the monotherapy group and 80% (12/15) in the combination group. On the basis of these data, the following assumptions were made:

- TGT monotherapy—ACR20 response rate=54%

- Combination therapy (TGTs+methotrexate, TGTs+hydroxychloroquine, and TGTs+leflunomide)—ACR20 response rate=80%

We consider this absolute difference of 26 percentage points to be clinically meaningful.

The sample size calculation was performed using PASS (Power Analysis and Sample Size) software (version 15; NCSS, LLC). The procedure selected was “chi-square tests” to compare multiple treatment groups with a common control while controlling the family-wise type I error rate.

Input parameters included the following: solving for sample size, degree of freedom 3; power 0.8; α=.05, and effect size (w=0.255098).

Consequently, the Dunnett procedure required 42 participants per group, resulting in a total sample size of 168 before accounting for dropouts. With an estimated dropout rate of 10%, the adjusted total sample size was approximately 188 participants, corresponding to approximately 47 participants per group.

### Data Management

Outcome assessors at each center recorded all study data. These assessors received standardized training prior to participant recruitment. The training covered procedures for accurately and promptly completing case report forms (CRFs) and electronic CRFs. Standard operating procedures were established and made available to investigators and assessors at each trial site.

Investigators were not permitted to modify or access data until enrollment, follow-up, and data collection had been completed for all participants.

### Statistical Analysis

The primary outcome—the ACR20 response rate at week 12—will be compared between each combination therapy group and the TGT monotherapy group using logistic regression. The study center will be included as a fixed effect to account for potential site differences.

Secondary outcomes (eg, DAS28-ESR and CDAI) will be analyzed using mixed-effects models for repeated measures. These models will include fixed effects for treatment, visit, treatment-by-visit interaction, baseline value, and study center, with participants included as a random intercept.

All primary analyses will be conducted on the intention-to-treat population, defined as all randomized participants who received at least 1 dose of the study drug. Missing data will be handled using multiple imputation under the missing-at-random assumption, generating 20 imputed datasets. Sensitivity analyses will be performed to evaluate the robustness of conclusions under different missing data assumptions.

Safety data, including adverse events, will be summarized descriptively by treatment group. Between-group comparisons of adverse event rates will be conducted using the Fisher exact test.

Rescue therapy with NSAIDs was permitted for intolerable joint pain, in line with ethical requirements and standard clinical practice for the management of RA. As pain and swelling are universal symptoms of active RA, the requirement for rescue analgesia reflects real-world treatment conditions and will not be statistically adjusted in the primary analysis. The use of rescue medication will be documented descriptively and reported by treatment group. If notable between-group differences in the frequency of rescue therapy are observed, exploratory sensitivity analyses will be conducted to assess their potential influence on efficacy outcomes.

All analyses will be performed using SAS (version 9.4) and R (version 4.4.s2; R Foundation for Statistical Computing). Between-group differences will be reported with 95% CIs. Statistical significance is set at α=.05 (2-sided).

### Quality Control

All investigators received specific training on trial objectives, protocols, treatment strategies, and quality control procedures before the trial commenced. All investigators involved in this study were rheumatologists with at least 5 years of clinical experience.

All trial documents, including screening forms, CRFs, and treatment records, are stored at the study site under locked conditions with restricted access.

Every 3 months, members of the quality monitoring team will conduct on-site quality control reviews at each participating site and generate reports on the overall quality of the study processes. Trial managers will hold regular meetings to discuss and address any issues identified during monitoring.

### Ethical Considerations

The Dongzhimen Hospital Ethics Committee of Beijing University of Chinese Medicine approved the study protocol (2024DZMEC-241). The trial is conducted in accordance with the Declaration of Helsinki and is registered with the Chinese Clinical Trial Registry.

Each participant was informed of the study’s purpose, potential risks, and benefits. The recruiting physician reviewed the written informed consent form with the participant. Participants did not receive any financial compensation for their involvement in this study, as the trial involved only marketed drugs with well-established safety and efficacy profiles, and no additional procedures beyond routine clinical practice were required.

In this study, personal identifying information was removed and replaced with a unique, nonidentifiable code. No personal information was transmitted, ensuring data confidentiality. The results of this study will be presented at selected conferences and scientific meetings and published in peer-reviewed journals. To promote transparency, the original dataset will be shared with external researchers upon reasonable request, beginning three years after the main results are published.

## Results

Recruitment began on September 1, 2024, and ended on December 31, 2025, with a total of 181 patients enrolled. Final data collection has been completed, and data cleaning is underway. Results are expected to be published by the end of 2026.

## Discussion

### Summary of Anticipated Findings

This trial is expected to provide robust evidence on the comparative efficacy and safety of TGT-based combination therapies in patients with moderately active RA. We hypothesize that all 3 TGT combination regimens (TGTs+methotrexate, TGTs+hydroxychloroquine, and TGTs+leflunomide) will result in significantly higher ACR20 response rates at week 12 than TGT monotherapy. These findings are expected to clarify the risk-benefit profile of these combinations and identify which regimen offers the most favorable balance of efficacy and tolerability for different patient subgroups.

### Comparison With Existing Evidence and Clinical Implications

The trial focuses on individuals with DAS28 scores between 3.2 and 5.1. This subgroup is targeted because early intervention with csDMARD combinations may prevent disease progression and reduce long-term disability. TGTs, a cornerstone of traditional Chinese medicine, have demonstrated efficacy comparable to methotrexate in monotherapy. However, their role in combination regimens remains poorly established [15,17,25]. This trial directly compares TGTs with 3 commonly used csDMARDs (methotrexate, leflunomide, and hydroxychloroquine), providing insights into synergistic effects, safety trade-offs, and optimal dosing strategies. The 12-week intervention period is designed to detect early treatment responses, which are critical for patients requiring rapid symptom control and for clinicians making timely therapeutic adjustments. Additionally, excluding participants with reproductive plans ensures a focus on the safety profile of TGTs in populations less vulnerable to their known gonadal toxicity, thereby refining the risk-benefit assessment of TGTs in clinical practice.

The results of this trial could reshape RA treatment paradigms, especially in settings with limited resources where biologic agents are unavailable. If TGT-based combination therapy is shown to be noninferior or superior to monotherapy, it could become a cost-effective initial treatment strategy, reducing the need for expensive biologics. Furthermore, the results of this study will provide evidence-based support for personalized treatment decisions involving TGT combination regimens.

### Strengths and Limitations

Exclusion of patients with reproductive potential may discourage recruitment, resulting in an eligible population composed predominantly of middle-aged and older individuals. To mitigate this issue, the trial was supported by a social media campaign that highlighted the study’s focus on safety in the nonreproductive population.

Compliance is another concern, as the risk of gastrointestinal side effects and hepatotoxicity associated with TGTs may lead to early discontinuation. To address this, active measures were implemented, including standardized patient education, routine monitoring of liver enzymes, protocol-based dose adjustments, aggressive follow-up, intensive training of participating investigators, and the use of digital joint counting tools to standardize scores.

Finally, because neither the investigators nor participants were blinded, and only outcome assessors and statistical analysts were blinded to group allocation, there is a potential risk of bias in the results.

### Conclusions

This prospective, multicenter, open-label RCT will provide robust evidence on the efficacy and safety of TGTs in combination with csDMARDs for the treatment of RA with moderate disease activity.

## Abbreviations

ACR: American College of Rheumatology
ACR20: American College of Rheumatology 20% improvement
bDMARD: biologic disease-modifying antirheumatic drug
CDAI: Clinical Disease Activity Index
CRF: case report form
CRP: C-reactive protein
csDMARD: conventional synthetic disease-modifying antirheumatic drug
CTCAE: Common Terminology Criteria for Adverse Events
DAS28: Disease Activity Score-28
DAS28-CRP: Disease Activity Score-28 using C-reactive protein
DAS28-ESR: Disease Activity Score-28 using erythrocyte sedimentation rate
ESR: erythrocyte sedimentation rate
EULAR: European Alliance of Associations for Rheumatology
HAQ-DI: Health Assessment Questionnaire Disability Index
IL-6: interleukin-6
MDGA: medical doctor global assessment
NSAID: nonsteroidal anti-inflammatory drug
PGA: patient global assessment
RA: rheumatoid arthritis
RCT: randomized controlled trial
SDAI: Simplified Disease Activity Index
SF-36: 36-Item Short Form Health Survey
SJC: swollen joint count
TGT: Tripterygium glycoside tablet
TJC: tender joint count
TNF-α: tumor necrosis factor-α
tsDMARD: targeted synthetic disease-modifying antirheumatic drug
VAS: visual analog scale
